# 

**Published:** 1858-06

**Authors:** 


### TWELFTH VOLUME

OF THE

### DENTAL REGISTER OF THE WEST.

EDITED BY

### J. TAFT and G. WATT.

TERMS: - - - - - - - $3 PER ANNUM, IN ADVANCE.

### Advertisements Inserted as follows:

One page, per annum,............................................$25 00
One-half page, per annum............................................15 00
Quarter page, per annum,............................................10 00

Shorter periods than one year, by special arrangement.

☞Contributions to the pages of the *Register* respectfully solicited.

Exchanges and Correspondents will please address "Dental Register," or "Taft & Watt, Cincinnati, Ohio."

